# Research Progress on Regulatory T Cells in Acute Kidney Injury

**DOI:** 10.1155/2015/174164

**Published:** 2015-07-26

**Authors:** Yamei Wang, Yuhong Tao

**Affiliations:** ^1^Department of Pediatrics, West China Second University Hospital, Sichuan University, Chengdu 610041, China; ^2^Key Laboratory of Birth Defects and Related Diseases of Women and Children (Sichuan University), Ministry of Education, Chengdu 610041, China

## Abstract

Immune inflammation is crucial in mediating acute kidney injury (AKI). Immune cells of both the innate and adaptive immune systems substantially contribute to overall renal damage in AKI. Regulatory T cells (Tregs) are key regulator of immunological function and have been demonstrated to ameliorate injury in several murine experimental models of renal inflammation. Recent studies have illuminated the renal-protective function of Tregs in AKI. Tregs appear to exert beneficial effects in both the acute injury phase and the recovery phase of AKI. Additionally, Tregs-based immunotherapy may represent a promising approach to ameliorate AKI and promote recovery from AKI. This review will highlight the recent insights into the role of Tregs and their therapeutic potential in AKI.

## 1. Introduction

Acute kidney injury (AKI) is caused by multiple etiologies that lead to renal dysfunction within a short period of time. Ischaemia reperfusion injury (IRI), nephrotoxic agents, and sepsis are among the major causes of AKI. AKI occurs in ~5% of hospitalized patients or 30% of critically ill patients, with detrimental consequences in terms of morbidity and mortality [1, 2]. Additionally, AKI increases the likelihood of developing chronic kidney disease and end-stage renal disease [3, 4]. Despite remarkable advances in blood purification, AKI remains to be a significant challenge that lacks specific tools to reduce kidney damage and promote kidney repair.

The pathogenesis of AKI is complex. Previous studies have revealed that immune inflammation is crucial in mediating AKI [5]. Immune cells of both the innate and adaptive immune systems, including dendritic cells (DCs), natural killer T cells, T and B lymphocytes, neutrophils, and macrophages, are well known for their participation in early injury [6]. Therefore, control of kidney inflammation can significantly reduce kidney damage in AKI [7, 8]. Removing, inhibiting, or antagonizing neutrophils, macrophages, T cells, and B lymphocytes have been shown to suppress renal inflammation and protect* in vivo* AKI models to varying degrees [9].

Regulatory T cells (Tregs) are a subset of CD4^+^T cells expressing the IL-2 receptor (CD25) and Forkhead Box P3 (Foxp3), a transcriptional factor that regulates the immunosuppressive activity of Tregs. Foxp3^+^Tregs account for approximately 2% of the total number of mononuclear cells in the normal kidney [6]. Tregs have inhibitory roles in various kidney diseases that include nephrotic syndrome, lupus nephritis, diabetic nephropathy, hypertensive renal injury, and other kidney diseases [10]. Recently, some studies have indicated that Tregs are protective and have become a potential target of AKI immunotherapy. In this review, we examine the research progress of Tregs in AKI.

## 2. Tregs Overview

Tregs are a developmentally and functionally distinct T cell subpopulation that is engaged in sustaining immunological self-tolerance and homeostasis [11, 12]. Natural Tregs (nTregs) are derived centrally in the thymus in response to self-antigens and regulate peripheral tolerance. Inducible Tregs (iTregs) are induced in the periphery from naïve T cells upon antigenic stimulation in the presence of transforming growth factor-*β* (TGF-*β*) and IL-2. Activation and migration of Tregs into tissue are critical for the control of inflammation [13]. Like conventional T cells, Tregs require T cell receptor stimulation and costimulation for activation [14]. Circulating and tissue iTregs numbers depend on anatomic location and the specific inflammatory environment. Constitutive expression of adhesion molecules (e.g., integrin *α*E, CD62L, CD44, and selectin ligands) and chemokine receptors (e.g., CCR2, CCR4, CCR5, CCR6, CCR7, and chemokine C-X-C motif receptor 3) [15–17] mediate the migration of Tregs into the inflammation site [18, 19], where Tregs suppress the innate and adaptive immune response through contact-dependent and soluble mediators.

**Figure 1:**
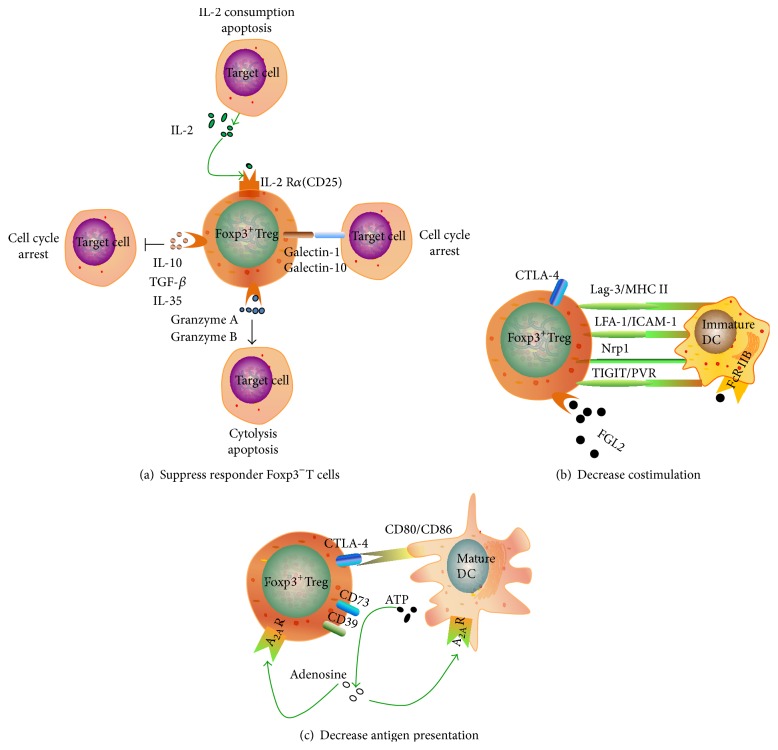
The mechanisms by which Tregs suppress the immune response. The main mechanisms include those that target Foxp3^−^T cells (cell cycle arrest, IL-2 consumption, apoptosis, and cytolysis) and those that primarily target dendritic cells (decreased costimulation or decreased antigen presentation).

First, Tregs may target Foxp3^−^T cells (Figure 1(a)). Tregs secrete suppressor cytokines (e.g., IL-10, TGF-*β*, and IL-35) that can directly result in inhibiting the function of Foxp3^−^T cells and cell cycle arrest [20]. In addition, Tregs suppress the proliferation of effector T cells by upregulating the expression of CD25 [21] and competing with effector T cells to deplete IL-2 [22], which ultimately induces apoptosis of effector T cells via a tumor necrosis factor-related apoptosis-inducing ligand [23]. Activated Tregs also express other soluble mediators, such as galectin-1, galectin-10 [24, 25], granzyme A, and granzyme B [26, 27], which can interact with effector T cells, resulting in cell cycle arrest, apoptosis, or cytosis.

Second, Tregs may primarily target DCs to decrease costimulation or antigen presentation (Figures 1(b) and 1(c)). Most Tregs express the cytotoxic T cell surface-associated antigen 4 (CTLA-4) on their surface [28, 29]. Tregs maintain close interaction with immature DCs through lymphocyte activation gene-3 (Lag-3)/MHC II molecules [30], leukocyte function-associated antigen-1 (LFA-1)/intercellular adhesion molecule 1 (ICAM-1) [31], and neuropilin-1 (Nrp1) [32] on their cell surface and rely on CTLA-4 to inhibit the maturation of DCs [33]. Lag-3 on Tregs can interact with MHC II molecules on immature DCs and results in an inhibitory signal that suppresses DC maturation and immunostimulatory capacity. Nrp-1 promotes long interactions between Tregs and immature DCs and restricts access of the effector T cells to DCs. Tregs expressing the T cell immunoreceptor with Ig and ITIM domains (TIGIT) are combined with the poliovirus receptor (PVR) on the surface of DCs, which induce DCs to produce IL-10 and TGF-*β* for immune suppression [34]. Tregs secreting fibrinogen-like protein 2 (FGL2) combined with low affinity Fc receptor type IIB (FcR IIB) inhibit the maturation of DCs [35]. In FGL2 knockout (KO) mice, the number of DCs increased with the stimulation of lipopolysaccharide, upregulation of CD80 and MHC II molecules, and the subsequent increase in the number of Tregs [36]. CTLA-4 on the surface of Tregs prevents the upregulation of CD80/CD86 on mature DCs and decreases antigen presentation [37] (Figure 1(c)). The ATP released from damaged cells induces the activation and inflammation of DCs. Tregs express high levels of CD39 (ectonucleoside triphosphate dephosphorylase 1) and CD73 (ecto-5′-nucleotidase) that convert extracellular ATP to adenosine [38], which has anti-inflammatory effects through adenosine 2A receptors (A_2A_Rs). Tregs generated adenosine signal through A_2A_Rs on inflammatory cells and in an autocrine manner on Tregs themselves [39]. In addition, activation of A_2A_Rs on mature DCs resulted in enhanced CD54, CD80, MHC I molecules, and HLA-DR molecule expression as well as a dose-dependent inhibition of TNF-*α* and IL-12 and augmentation of IL-10 secretion [40].

## 3. Roles of Tregs in AKI

### 3.1. Tregs in Ischemic AKI

Kinsey et al. [41] used an anti-CD25 monoclonal antibody (PC61) to partially deplete Tregs in an* in vivo *mouse model 5 days prior to kidney IRI. Twenty-four hours after renal IRI, nephritis, tubular necrosis, and renal function declined in PC61-treated mice, changes that were significantly greater than those observed in control mice. Reducing the number of Tregs resulted in more neutrophils, macrophages, and innate immune system cytokines (i.e., IL-6, tumor necrosis factor-*α* (TNF-*α*), and TGF-*β*) in the kidney after IRI but did not affect CD4^+^T cells or B cells. Kinsey et al. [41] also performed adoptive transfer of lymph node cells from wild type (WT) mice or Scurfy mice (Foxp3-deficient mice) into RAG-1 KO mice (T cell and B cell-deficient mice) to generate mice with and without Foxp3^+^Tregs, respectively. The Foxp3^+^Treg-deficient mice accumulated a higher number of inflammatory leukocytes after renal IRI than mice containing Tregs. Moreover, this increased renal damage was reversed by isolated WT Tregs transfer to the Scurfy lymph node cells in the RAG-1 KO model. Therefore, Tregs regulated the inflammatory responses of innate immunity at the early stage of renal IRI and alleviated kidney damage.

Monteiro et al. [42] used PC61 prior to renal IRI in a mouse model. In this study, seventy-two hours after renal IRI the kidney function declined, and kidney damage was markedly exacerbated, which suggested that Tregs alleviate kidney damage. Gandolfo et al. [43] revealed that Tregs depletion using PC61, starting one day after renal IRI, exacerbated renal tubular damage, reduced tubular proliferation, and increased cytokine production by infiltrating T cells on day 3 and increased TNF-*α* generation by CD4^+^T cells on day 10. However, adoptive transfer of Tregs on day 1 after IRI resulted in reduced production of IFN-*γ* by CD4^+^T cells on day 3 and improved repair and reduced the generation of proinflammatory cytokines by day 10 [43]. Furthermore, during the repair phase, administration of mycophenolate mofetil reduced the total number of kidney mononuclear cells and decreased the population of Tregs, which inhibited recovery from renal IRI [44]. Jun et al. [45] also showed that CD4^+^CD25^high^CD127^low^ Tregs expansion promoted kidney repair, and PC61 treatment aggravated kidney damage in a mouse renal IRI model. Collectively, Tregs traffic to the injured kidney may promote repair from renal IRI.

Ischemic preconditioning (IPC) is a stronger measure of protecting the kidneys from IRI [46]. IPC is partially mediated by Tregs and significantly inhibits the accumulation of neutrophils and macrophages, tubular necrosis, and loss of kidney function caused by a subsequent renal IRI one week later [47]. PC61 treatment before the second ischemia in IPC mice resulted in a decrease [48] or complete inhibition of the renal-protective effect of IPC [49]. Thus, Tregs might be involved in IPC-induced renal protection.

### 3.2. Tregs in Nephrotoxic AKI

The renoprotective effects of Tregs have also been found in models of nephrotoxic AKI. In experimental murine AKI induced by cisplatin [50], the adoptive transfer of Tregs attenuated renal injury and decreased macrophage infiltration in both (mature-T-cell-deficient)* Foxn1* ^*nu/nu*^ mice and WT Balb/c mice. Consistently, Tregs depletion with PC61 before cisplatin administration resulted in worse renal function and tissue injury.

### 3.3. Tregs in Septic AKI

Sepsis is considered an excessive systemic inflammation. However, the pathogenesis of septic acute kidney injury is thought to be different from that of ischemia/reperfusion induced AKI. Furthermore, the inhibition of inflammation has been shown to have no effect on sepsis. In a mouse model of cecal ligation and puncture- (CLP-) induced sepsis, septic AKI was associated with an increase in IL-10 and increased Tregs [51]. In contrast to renal IRI, depletion of Tregs before CLP resulted in renoprotection [51]. Cho et al. [52] also showed that increases in serum soluble CD25 and IL-10 in patients with septic AKI were strongly associated with immunosuppression. Hence, Tregs may contribute to septic AKI.

## 4. The Action Mechanism of Tregs in AKI

Although Tregs utilize various mechanisms to suppress renal inflammation in AKI, there are many questions that need to be answered regarding the action mechanism of Tregs in AKI. To date, most of the mechanisms depicted above still have not been investigated in AKI models. IL-10 is a potent anti-inflammatory cytokine that inhibits inflammatory pathways [53]. Kinsey et al. [41] found that RAG-1 KO mice exposed to more prolonged renal IRI were protected by the adoptive transfer of WT Tregs. However, this protective effect was lost if IL-10 KO Tregs were administered, thus implicating IL-10 as a key mediator of Tregs protection. In the study of Gandolfo et al. [43], the role of IL-10 production by Tregs remained unclear, as IL-10 expression was markedly increased in the aftermath of Tregs depletion in the recovery phase of renal IRI. Kinsey et al. [54] demonstrated that adoptively transferred WT Tregs protected WT mice from kidney IRI, but CD73-deficient Tregs or A_2A_Rs-deficient Tregs led to the inhibition of Tregs function. A_2A_Rs activation can enhance the expression of programmed cell death 1 (PD-1) on the cell surface of Tregs. However, the blockade of PD-1, prior to adoptive transfer, negates their ability to protect against ischemic AKI [54]. Additionally, both PD-1 ligands (PD-L1 and PD-L2) protect the kidney from IRI [55]. Collectively, these results demonstrate that IL-10, adenosine, A_2A_Rs, and PD-1 are required for Tregs to suppress immune responses in renal IRI. In addition, both the CTLA-4 and Tregs are essential for the control of immune homeostasis. Tregs commonly use CTLA-4 to affect suppression [56]. However, previous research has shown that the use of an anti-CTLA-4 antibody treatment counteracted the protection from renal IRI induced by N,N-dimethylsphingosine (DMS) [57], suggesting that CTLA-4 is also involved in the protective effects of Tregs in AKI.

## 5. New Treatment Strategies for AKI Using Tregs

### 5.1. Adoptive Transfer of CD4^+^CD25^+^Tregs

Tregs are the new targets for AKI immunotherapy [8, 58], and the most direct method is the adoptive transfer of Tregs [59]. Previous studies have indicated that autologous and donor-derived Tregs have similar protective effects on animal models with intestinal IRI [59], stroke [60], and burns [61]. Tregs adoptive immunotherapy can also alleviate kidney damage in animal models with adriamycin-induced nephropathy [62], antiglomerular basement membrane nephritis [63], and lupus nephritis [64]. Prior to renal ischemia [41] and cisplatin treatment [50], the transfer of freshly isolated CD4^+^CD25^+^Tregs (1 × 10^6^ cells/mouse) from a normal WT mouse spleen to an AKI mouse model alleviated kidney damage and improved the survival rate of the AKI mice. In addition, the transfer of freshly isolated CD4^+^CD25^+^Tregs (1 × 10^6^ cells/mouse) from a normal WT mouse spleen to animals 24 hours after renal IRI increased the number of Tregs in the kidneys. A reduction in the production of TNF-*α* and IFN-*γ* from effector T cells in the kidneys, improvement in tubular necrosis score, and acceleration of kidney repair were also observed. Although none of these reports have refuted the protective effect of adoptive transfer of Tregs in an aseptic AKI model [41, 43, 47, 50, 54], various studies still dispute the therapeutic efficacy of adoptive transfer of Tregs [65].

Because adoptive transfer therapy requires a high number of Tregs, the* in vitro* amplification and induced production of Tregs using other methods is necessary. CD127 was recently discovered as an antigen that is associated with Tregs, although it is expressed at relatively low levels [66]. After magnetically activated cell sorting of CD4^+^CD25^high^Foxp3^+^T cells, CD127 was used as a molecular marker for screening cells with highly expressed CD127 to significantly improve the purity of Tregs [67]. Application of the anti-CD3/CD28 monoclonal antibody-coated magnetic beads in the presence of 1,000–2,000 units of recombinant IL-2 effectively amplifies the CD4^+^CD25^+^Tregs and inhibits T cell proliferation and cytokine production [59]. The addition of rapamycin and Accutane during* in vitro *cell culture also stabilizes the expression of Foxp3 in Tregs [68].

The current treatments of adoptive Tregs are mainly restricted to animal models and have not been confirmed in human AKI. However, Tregs adoptive immunotherapy in certain diseases has reached the clinical trial stage [69–71]. In 2011, Brunstein et al. [72] intravenously injected patients with* in vitro* amplified Tregs (3 × 10^6^ cells/kg) from umbilical cord blood after stem cell transplantation. As a result, a significantly reduced incidence of graft-versus-host disease was observed, whereas the risks of infection, primary disease recurrence, and early death did not increase. Thus, these studies have served as the foundation for Tregs adoptive immunotherapy in human AKI.

One of the most challenging problems of Tregs adoptive immunotherapy involves* in vitro *amplification of Tregs in normal T cells. Studies on the plasticity of T cell differentiation by Zhou et al. [73] prompted us to pay more attention to this possibility. Komatsu et al. [74] indicated that most adoptive Tregs could maintain their immunosuppressive activity. However, a small number of adoptive Tregs show a depletion of Foxp3 and a subsequent change to normal T cells, ultimately resulting in the restoration of pathogenicity. To date, the application of magnetic activated cell sorting to sort the enriched Tregs cannot completely remove the effector T cells, which results in a mixture of a large number of T lymphocytes in the* in vitro* amplified Tregs. Thus, the preservation of* in vitro* amplified Tregs has become a significant concern among researchers [75].

### 5.2. Targeting of Intrinsic Tregs

An alternative approach is to target intrinsic Tregs to enhance Tregs numbers, trafficking, or activity. Recently, several pharmaceutical agents that target intrinsic Tregs have been utilized in AKI models with encouraging results (Table 1). Bee venom and its constituent phospholipase A2 are capable of modulating Tregs in the spleens of mice [76]. Recently, Kim et al. [77] reported that bee venom had protective effects on cisplatin-induced nephrotoxicity in mice, at least in part, through increasing Tregs numbers and enhancing Tregs trafficking without a large influence on the antitumor effects of cisplatin. Phospholipase A2 also prevented inflammatory responses in cisplatin-induced AKI by modulating Tregs and IL-10 through the CD206 mannose receptor [78]. Lai et al. [57] demonstrated that DMS, a naturally occurring sphingosine derivative, allowed Tregs to rapidly and transiently migrate to the kidneys, and pretreatment with DMS provided renoprotection in IRI. This DMS-induced renoprotection was abolished by the administration of agents that suppress Tregs or by anti-CTLA-4 or anti-CD45 monoclonal antibodies. However, another sphingosine kinase inhibitor did not produce similar protection. The sphingosine-1-phosphate analog (FTY720) is a new synthetic immunosuppressant that involves the structural transformation of the active ingredient of* Cordyceps* extract, myriocin. FTY720 exerts its immunosuppressive effects by reacting with the sphingosine-1-phosphate receptor on the cell surface. Treatment with FTY720 could attenuate renal IRI and reduce inflammation. The beneficial effects of FTY720 in renal IRI may be partially mediated by increasing Tregs activity [79]. The injection of IL-2/IL-2 monoclonal antibody (mAb) complexes into mice results in a 10-fold* in vivo *Tregs expansion [80]. Kim et al. [81] also showed that the IL-2/anti-IL-2 complexes administered before bilateral renal IRI, induced Tregs expansion in both the spleen and kidney, improved renal function, and attenuated renal injury and apoptosis after IRI. The aryl hydrocarbon receptor has emerged as a major modulator of inflammatory processes. The aryl hydrocarbon receptor agonist, leflunomide, increased Tregs and IL-10-positive cells but reduced IL-17-expressing and IL-23-expressing cells in both the peripheral blood and kidney cells in renal IRI mice [82]. Mesenchymal stem cells (MSCs) induced differentiation of naïve T cells through paracrine mechanisms [83] and promoted the* in vivo *amplification of Tregs [84, 85]. MSCs ameliorate renal IRI by inducing regulatory T cells through interactions with splenocytes. PC61 treatment or splenectomy reduced the renal-protective effect of MSCs [86]. miR-26a has been reported to play functions in cellular differentiation, cell growth, cell apoptosis, and metastasis [87]. Liang et al. [88] reported that miR-26a treatment induced significant expansion of Foxp3^+^Tregs in both the spleen and kidneys on day 10 after IRI and attenuated renal IRI. Thus, these studies suggest that targeting intrinsic Tregs may be a promising option for AKI.

There are other therapeutic agents, such as ATL1222^b^ (A_2A_R agonist) [54], aspirin [89], and gardiquimod [90], which have been shown to enhance Tregs* ex vivo* or* in vitro*. However, none of them have been investigated with* in vivo* AKI models. With the development of assay platforms to screen for the Tregs modulating potential of pharmacological compounds [91], numerous novel agents will likely be found to boost Tregs number or activity* in vivo*.

## 6. Conclusions

Overall, Tregs have a renal-protective function through the suppression of renal inflammation, which is critical for blocking renal injury and/or promoting recovery from AKI. Based on the research in animal models, Tregs and their associated factors have been considered as potential therapeutic targets for AKI immunotherapy. An increasing understanding of Tregs' functional mechanisms in AKI will lead to a number of clinical trials on the discovery and development of new Tregs-oriented therapies.

## Figures and Tables

**Table 1:** Therapeutic strategies for AKI based on intrinsic Tregs.

Reference	Animal model	Agent	*In vivo* effect
Kim et al. [77]	Cisplatin-induced AKI	Bee venom	↑ Tregs numbers ↑ Tregs trafficking
Lai et al. [57]	Ischemic AKI	Dimethyl sphingosine	↑ Tregs trafficking
Kim et al. [79]	Ischemic AKI	FTY720	↑ Tregs numbers
Kim et al. [81]	Ischemic AKI	IL-2/anti-IL-2 complexes	↑ Tregs numbers
Baban et al. [82]	Ischemic AKI	Leflunomide	↑ Tregs numbers
Hu et al. [86]	Ischemic AKI	Mesenchymal stem cells	↑ Tregs numbers
Liang et al. [88]	Ischemic AKI	miR-26a	↑ Tregs numbers

## Abbreviations

A_2A_R: Adenosine 2a receptors
AKI: Acute kidney injury
CLP: Cecal ligation and puncture
CTLA-4: Cytotoxic T cell surface-associated antigen 4
DCs: Dendritic cells
DMS: N,N-Dimethylsphingosine
FcR IIB: Fc receptor type IIB
FGL2: Fibrinogen-like protein 2
Foxp3: Forkhead Box P3
ICAM-1: Intercellular adhesion molecule 1
IPC: Ischemic preconditioning
IRI: Ischaemia reperfusion injury
iTregs: Inducible Tregs
KO: Knockout
Lag-3: Lymphocyte activation gene-3
LFA-1: Leukocyte function-associated antigen-1
MSCs: Mesenchymal stem cells
Nrp1: Neuropilin-1
nTregs: Natural Tregs
PD-1: Programmed cell death 1
PVR: Poliovirus receptor
TGF-*β*: Transforming growth factor-*β*
TIGIT: T cell immunoreceptor with Ig and ITIM domains
TNF-*α*: Tumor necrosis factor-*α*
Tregs: Regulatory T cells
WT: Wild type.
